# Shared decision-making needs, barriers, and facilitators of patients with newly diagnosed advanced cancer in the hospital: a multi-level, mixed-methods study

**DOI:** 10.1007/s00520-024-08515-1

**Published:** 2024-04-29

**Authors:** Kevin R. Reyes, Paul Wong, Mary Petrofsky, Annie Dai, Alyson Pelayo, Sam Brondfield, Daniel H. Kwon

**Affiliations:** 1 Department of Medicine, University of California, San Francisco (UCSF), San Francisco, CA USA; 2 UCSF Helen Diller Family Comprehensive Cancer Center, University of California, San Francisco (UCSF), San Francisco, CA USA

**Keywords:** Shared decision-making, Inpatient, Medical oncology, Mixed-methods study, Patient education, Counseling, Cancer

## Abstract

**Purpose:**

Little is known about the shared decision-making (SDM) needs, barriers, and facilitators of patients with newly diagnosed advanced cancer in the hospital. Understanding this may improve SDM and cancer care quality in this vulnerable population.

**Methods:**

A single-site, mixed-methods study of hospitalized patients with newly diagnosed advanced cancer, caregivers, and oncologists was conducted. After discharge, patient ± caregiver semi-structured interviews exploring SDM needs, barriers, and facilitators regarding their most important upcoming cancer-related decision were conducted. Oncologists were surveyed about patient knowledge and SDM needs using closed- and open-ended questions, respectively. Thematic analysis was performed for qualitative data with a focus on themes unique to or amplified by hospitalization. Descriptive statistics and the Chi-squared test were performed for quantitative data.

**Results:**

Patients and caregivers reported high SDM needs surrounding treatment and prognostic information, leading to decisional conflict. Eight themes emerged: anticipated cancer treatment decisions, variable control preferences in decision-making, high cancer-related information needs and uncertainty, barriers and facilitators to information gathering during and post hospitalization, and decision-making facilitators. Among 32 oncologists, most (56%) reported patients were poorly informed about treatment and prognosis. Oncologists reported variable expectations about patient knowledge after hospitalization, facilitators to patient decision-making, and patient uncertainty while awaiting an outpatient oncologist appointment.

**Conclusion:**

Patients newly diagnosed with advanced cancer in the hospital have high SDM needs and experience decisional conflict. This may be due to barriers unique to or exacerbated by hospitalization. Further research is needed to develop strategies to address these barriers and enhance the facilitators identified in this study.

**Supplementary Information:**

The online version contains supplementary material available at 10.1007/s00520-024-08515-1.

## Introduction

Shared decision-making (SDM) is a collaborative process in which patients and providers partner to make informed decisions in line with patients’ needs and priorities [1]. This entails (1) informing patients that options are available, (2) providing more detailed information on the options, and (3) supporting patients in exploring their preferences and deciding what is best for them [1]. SDM is imperative in preserving patient autonomy and delivering high-quality cancer care [2, 3]. However, for patients newly diagnosed with advanced cancer, SDM is challenging due to the amount and complexity of information and undesirable emotions associated with the diagnosis [4, 5].

Prior studies have found that patients diagnosed with cancer and their caregivers have a high level of unmet information needs, particularly surrounding treatment benefits and side effects [4, 6]. However, there is a limited understanding about the information and decisional needs of patients who are diagnosed with advanced cancer in the hospital as they await establishing outpatient oncology care. Hospitalized patients may face greater information needs due to unique barriers related to hospitalization, such as acute illness, inpatient-outpatient transitions, and worse survival outcomes [7]. Understanding these needs and barriers would inform potential inpatient interventions to help patients engage in SDM in the clinic, thereby enhancing the overall quality of cancer care provided to this vulnerable population.

Here, we conducted a mixed-methods study to understand the information and decisional needs of these patients. We also explored barriers/facilitators to these needs, particularly those related to their hospitalization. We focused on patient information needs, as opposed to the deliberation of preferences with one’s physician that is crucial in SDM, as the latter cannot be observed in hospitalized patients who have yet to meet their outpatient oncologist.

## Methods

### Study design

A convergent-parallel mixed-methods study of patients newly diagnosed with an advanced solid malignancy in the hospital, their caregivers, and outpatient oncologists was conducted at the University of California, San Francisco (UCSF) from 6/2022–11/2022. A mixed-methods approach was chosen to obtain rich, contextualized insights and validate findings across multiple methods. Patients, caregivers, and oncologists were selected because they play important roles in patient decision-making based on the Ottawa Decision Support Framework (ODSF) [8, 9]. ODSF conceptualizes the support needed by patients, families, and providers for difficult decisions. For this study about patients newly diagnosed with advanced cancer in the hospital, this decision was the most important anticipated cancer-related decision identified by these patients [8, 9].

Patients were consecutively sampled in person among inpatients for whom medical oncology consultation was requested. Eligibility criteria included (1) English-speaking adults, (2) clinical suspicion for newly diagnosed unresectable/metastatic non-hematologic malignancy (biopsy-confirmed or in-process with preliminary results), (3) no inpatient systemic therapy, and (4) no plan for hospice care. Patients had been told of their cancer diagnosis by a member of either the primary or consulting oncology team prior to being approached by the study team. Patients had the option of inviting a caregiver, who was then approached by the study team to participate in dyadic interviews. All medical oncologists who provide ambulatory care for patients with non-hematologic cancers at UCSF were approached. Patients and oncologists were reimbursed with $20 and $10 gift cards, respectively; caregivers were not reimbursed. The COREQ checklist for reporting qualitative research was followed (Supplementary Table 1) [10].

### Data collection

#### Patient characteristics

To characterize the patient sample, patients were administered a survey at discharge with questions about demographics, whether they know their cancer type and stage, and decisional conflict using the 16-item decisional conflict scale (Supplementary Fig. 1) [9].

#### Patients and caregivers: qualitative data collection

Semi-structured interviews with patients ± caregivers were conducted 7–14 days post-discharge. An interview guide was developed and pilot-tested using the Ottawa Hospital Research Institute’s Decisional Needs Assessment, a data collection strategy based on the ODSF to identify the needs of patients to make better decisions (Supplementary Fig. 2) [11]. Questions were about patients’ understanding of their cancer, their most important upcoming cancer-related decision, the information and decisional support needed to make this decision, and barriers/facilitators to these needs. Interviews were conducted by telephone or virtual conference, audio-recorded, and transcribed by two co-authors. The sample size for the initial analysis was set at 8, and participants were interviewed until saturation was met, defined as no new themes in two consecutive interviews [12].

#### Oncologists: quantitative and qualitative data collection

Oncologists were e-mailed a pilot-tested survey that instructed them to recall the last appointment they had with a patient who was newly diagnosed with advanced cancer in the hospital and then asked how well-informed the patient was about cancer type, stage, treatment, and prognosis (4 items, 5-point Likert scale). The survey also included three open-ended questions about (1) cancer-related information patients should know before their initial appointment, (2) recommended cancer-related resources, and (3) suggestions for inpatient oncologists to facilitate SDM for patients at their future outpatient oncology visit (Supplementary Fig. 3).

### Data analysis

#### Quantitative data

Quantitative data were summarized using descriptive statistics. The proportion of oncologists who reported their patient was very/somewhat poorly informed was compared pairwise for each information domain using the Chi-squared test, with “cancer type” as the reference domain. Statistical analyses were conducted using R 4.2.1 software with *p* < 0.05 deemed statistically significant.

#### Qualitative data

Constant comparison thematic analysis was conducted using a deductive approach while remaining open to new themes. Analysis was focused on themes that were unique to or amplified by hospitalization. An initial codebook was created using the interview guide and updated during the coding process using constant comparison. Transcripts were first coded by K.R.R. or P.W. using ATLAS.ti; then, secondary coding was performed by the other co-author. Discrepancies were negotiated to a consensus. Unresolvable discrepancies were addressed by D.H.K. Codes were then categorized into themes and subthemes by K.R.R. and D.H.K.

#### Quantitative–qualitative data integration

Quantitative and qualitative results were reported separately. During interpretation, K.R.R. and D.H.K conducted methodological triangulation by comparing qualitative patient and caregiver interview data with quantitative and qualitative oncologist survey data to look for similarities and disagreements across findings from the different methods to gain a more complete understanding. Interpretations were described in the Discussion through a weaving narrative approach, in which quantitative and qualitative results were reported on a concept-by-concept basis [13].

## Results

### Patient and caregiver characteristics

Saturation was met after 12 interviews, which includes 12 patients and 4 caregivers. Eighteen patients were approached to yield this sample size (response rate 67%). Median age was 72.5 years. Seven (58%) identified as male and 5 (42%) female; 5 (42%) identified as Non-Hispanic White (Table 1). All caregivers were female; two were daughters (50%), one (25%) sister, and one (25%) wife. Nine (75%) patients reported knowing their cancer type, and five (42%) knew the stage. Eight (67%) patients had a decisional conflict scale score > 37.5 (scored 0–100), which correlates with decision delay or uncertainty [11].

**Table 1:** Patient characteristics

Characteristic	Total cohort (*N* = 12)
Age (years), median (range)	72.5 (64–81)
Gender identity	
Male	7 (58%)
Female	5 (42%)
Race/ethnicity	
Non-Hispanic White	5 (42%)
Black/African American	4 (33%)
Hispanic White	1 (8%)
Asian (Chinese)	2 (17%)
Cancer type	
Lung	6 (50%)
Colorectal	2 (17%)
Kidney	1 (8%)
Pancreas	1 (8%)
Pheochromocytoma	1 (8%)
Sarcoma	1 (5%)
Occupational status	
Employed	1 (8%)
Retired	8 (67%)
Disabled	2 (17%)
Other/self-employed	1 (8%)
Marital status	
Married	4 (33%)
Divorced	5 (42%)
Widowed	3 (25%)
Highest education level attained	
8–11 years	2 (17%)
High school graduate	1 (8%)
Some college	4 (33%)
College graduate	3 (25%)
Postgraduate	2 (17%)
Household income	
$0–$19,999	1 (5%)
$20,000–$34,999	2 (17%)
$35,000–$49,999	2 (7%)
$50,000–$74,999	1 (8%)
$75,000–$199,999	2 (17%)
≥ $200,000	3 (25%)
Not answered	1 (8%)
Household size	
1	5 (42%)
2	4 (33%)
3	1 (8%)
4	0 (0%)
5	2 (17%)
Cancer knowledge	
Knows cancer type	9 (75%)
Knows cancer stage	5 (42%)
Decisional conflict scale	
≤ 37.5	4 (33%)
> 37.5	8 (67%)

### Patient and caregiver: qualitative results

Table 2 illustrates the full thematic analysis. Themes are described below in narrative form below Table 2, with patient quotes indicated by “P” and caregiver quotes by “C.”

**Table 2:** Thematic analysis of semi-structured interviews with patients and their caregivers about information and decisional needs

Themes	Subtheme	Codes	Representative quote(s)
Anticipated cancer treatment decisions	Decisional conflict and uncertainty	Insufficient information about cancer to know what most anticipated decisions are	“[Anticipated decisions about the cancer] are what I gotta figure out. How can I know the future unless I know what the potential is?…I gotta know what it is before I can even think about it.” (P2)
		Treatment decision depends on pending test results	“[The treatment decision] depends on how the results are when we find out. And I will decide but, I will not decide before the results come out.” (P3)
		Difficulty with cancer decisions due to lack of understanding of cancer treatment effects	“You got to get the information and then you got to see what effect it’s going to have on your body and whatever chemicals or whatever they’re going to do. So, I mean, that’s a very challenging thing.” (P2)
	Types of cancer decisions	Deciding whether to undergo treatment or not	“Every morning I’m leaning towards doing nothing because of my life experience… I’ve already been through enough that I’m tired. And I’m talking emotionally. Half of me died when I lost my wife. And I only have a half left.” (P4)
		Deciding what type of treatment to receive	“I’d say the answer [to your question about anticipated decisions] is both. What type of treatment and where to pursue other [alternative] treatments. If I feel like something else could give me a better chance.” (P5)
		Deciding how aggressively to treat cancer	“Depending upon the findings, [I anticipate making a decision about] if I’m going to undertake aggressive action.” (P1)
Variable control preferences in decision-making	Control lies with patient	Patient maintains decision-making autonomy	“I will hold the last decision being made as to how I will live the rest of my life.” (P1)
	Control lies with caregiver	Caregiver makes decision for patient	“[Caregiver’s brother] and I have been making the decisions for her since she has been too sick to be able to.” (C1)
	Control lies with provider	Patient will follow healthcare team’s recommendation	“I’m just going to go by what the pros, what the facility recommends. So, mainly waiting for that initial meeting with the cancer doctor.” (P11)
High cancer-related information needs and uncertainty	Information needs are unknown and general lack of cancer knowledge	What information to know is unknown	“I mean the thing is, you don’t know what you don’t know.” (C2)
		Know nothing about cancer	“Not really, nothing [has been told to me about the cancer].” (P8)
	Nature of cancer unknown	Cancer diagnosis and type unknown	“We stayed in the hospital for four or five days and nothing happened. Nobody told us what it was and nobody told us we were going to start treatment. We’re just sitting there worrying we’re going to die because we’re not going to get treatment.” (C2) “And they’re not exactly sure where this cancer derived from necessarily. Those answers have not been concluded yet. The biopsy is still in progress.” (P1)
		Cancer stage and involved organs unknown	“If I had many cancers, many organs with cancer, it doesn’t make sense to get treatment. They didn’t tell me how many organs have cancer.” (P3)
		Cancer molecular testing results and implications unknown	“I have no information as to the lung cancer component and what the biomarkers are… I want to understand the implications for prognosis of each type of those markers. I know that presence or absence of some lead to opportunities for different therapies that are more or less successful.” (P5)
	Cancer treatment details unknown	Cancer treatment options unknown	“No, we don’t know [what options we have]. Surgery is not an option. So, we’re looking at chemotherapy. Hopefully immunotherapy. Hopefully targeted therapy. But specifics we don’t know.” (C2) “Well, I’m not 100% sure [about the treatment options]. I know we’re going to start out with radiation and then I think after the cancer group determines how severe the cancer is and how they’re going to go about treating it. I’m just waiting to hear from them. What options I have. So yeah, I’m just basically waiting on now.” (P11)
		Cancer treatment oncologist recommendations unknown	“I want to know about the radiation and I want to know about the chemo. And I want the doctor to tell me what is the best thing I can take.” (P8)
		Cancer treatment benefits unknown	“Effectiveness. Is it going to work for some amount? Is it going to keep me level a little bit? I gotta find out all that stuff.” (P2) “What are the benefits against the non benefits [of treatment]. What is the longevity and health in my life versus the non-longevity and non-health in my life.” (P6)
		Cancer treatment side effects unknown	“Being sent home and not knowing how you’re going to feel or what side effects there are and stuff like that [are barriers to treatment].” (C4)
		Cancer treatment logistics (duration, frequency, and location) unknown	“Well, we want to know what kind of treatment? How long? how many rounds? What is the pace?” (C2) “I think transportation. How much care is needed? Where? And what frequency of treatment visitations? All those types of things.” (P5)
		Cancer treatment urgency unknown	“But I don’t know the seriousness and of how soon I should be treated.” (P11)
	Prognosis unknown	Likelihood of cure unknown	“Have they found any cures that have completely cured the disease. Not just gave treatment, but something they cured.” (P6) “[It is important to know] if [the cancer is] curable. And for her to at least get what’s remaining in her life, make it the best possible. It’s never too late to take a walk in the beach. You know what I’m saying?” (C1)
		Survival time unknown	“Knowing what we’re facing at the time is something a bit more immediate or something that is going to be long and drawn out [would be helpful].” (P1)
		Post-operative recovery unknown	“How long will I be in the hospital after surgery [is the most important information to find out].” (P10)
	Cancer supportive care unknown	Symptom management unknown	“We need medication support. Antiemetics is standard. So, I’m not concerned about that. But when it’s refractory, what is available?” (C2) “I don’t know why they didn’t send her home with anything but Tylenol. But then again, that could be her choice of medication.” (C1)
	Cancer care delivery unknown	Reasons for delays in cancer diagnosis unknown	“Well, it would be helpful if we knew what was the delay in finding out what kind of cancer was told to us earlier. We just felt like why are we waiting? Why are we sitting around and one person’s telling us small cell and then nobody else tells us anything conflicting or dissenting? So you tend to go along with this small cell business. And then it turns out it’s not so. I think that could be improved. (C2) “They said [the cancer] started in the colon. But there’s something else because it went to the kidney. So, with that being said, we haven’t gotten the final report and I don’t know why they didn’t give us the final report before her discharge.” (C1)
Barriers to information gathering during hospitalization	Information content barriers from providers	Relaying complex information	“The last meeting I had [in the hospital] was with the oncologist…And it was three hours, which is really a long time. And he explained everything, and it’s complicated…In three hours, I couldn’t understand most of it.” (P4)
		Lack of information from providers	“Nothing yet [has been to told me to about the cancer].” (P2)
	Information flow barriers	Contradictory information between hospitals	“Well, [the hospital] read the tests [from the prior hospital] and they said, ‘No, that’s wrong. It’s not small cell. It’s non-small cell.’” (C2)
		Inability to identify cause of chief complaint and cancer in previous care facility	“My situation was identified immediately. Within hours versus when I went through the same in Las Vegas. They never determined what exactly was going on with me after taking scans or tests.” (P1)
		Dynamic, sometimes contradictory, information among hospital providers	“[I did not have enough information about the cancer to make a treatment decision] at that point because everybody was still learning. … And they say we’ll just keep passing the information as it comes to us. … And each day was a little bit different.” (P4) “[The hospitalization] wasn’t so pleasant because like I said earlier, we were told that they were thinking of a small cell. So that’s enough to frighten the bejesus out of us, right? So, we were just so, [Patient’s name] was like, totally lost in fear.” (C2)
		Many providers giving information	“Caregiver: When you had lots of different doctors visiting, did you know who all of them were and what they were asking you? Patient: Sort of. Some I might know; some I don’t know.” (C3 & P3)
		Lack of communication with family	“I wasn’t present. My brother was present. And I asked him what happened, what’s going on. I don’t get an answer from my brother.” (C1)
	Inexperienced cancer team barrier	Distress from receiving cancer diagnosis from inexperienced medical student	“I think when they came and told us small cell, it was a medical student. And I don’t know what year she is, but I don’t think she knew a whole lot. It’s just that I don’t think it was a good idea to send a medical student to come talk to us about the type of cancer that he may have. Especially when it was a situation where they had a hard time discerning what type of cell that is because of the necrosis and the inability to differentiate under the microscope.” (C2)
	Information processing barriers	Distress from cancer hinders information processing	“They did explain to her she had cancer. So, I think she’s probably in denial because she is unable to accept it…to find this out is crushing to her.” (C1) “Frightening. Different. I haven’t had any push of freewill to this point. That would be complicated to a normal person, okay. But because of the fear [coming from the cancer]…and having never had this type of treatment before, it seems a little complicated to me.” (P6)
		Acuity of malignancy and hospitalization hindered information processing	“[In the hospital], I was just in a lot of pain and under a lot of medication and had difficulty learning some new skills as to how to navigate this new body.” (P5)
Facilitators to information gathering during hospitalization	Information content facilitators from providers	Provider inviting family members to medical conversations	“It was reassuring to have your staff talk to us with [my wife and daughter] in there because they were thinking clear and two heads or three heads are better than one so that really made a difference.” (P11)
	Information flow facilitators	Rapid communication of test results	“[Provider] let us know right away anything she knows. She will let us know right away what the test results are –– calling me and she would tell me and [patient name] what’s happening. It is reassuring to have someone care about you so much.” (C2)
		Availability of providers of medical information	What we didn’t understand they would … send us, phone info or emails. And so the communication part of it was covered. (P11)
	Caregiver facilitators	Caregiver explaining information	“Without our sister being able to explain all of the tests, I think it would have been a lot more difficult…Our sister is a doctor and is able to read her chart. She can relay that information, and that helped a lot.” (C3) “No [information that I’ve received has been confusing] because I’ve got my daughter and she’s brilliant…She’s my care provider and she’s got medical training… she has [been able to translate what the doctor has been saying for me].” (P10)
		Caregiver note-taking	“My daughter was the one taking notes.” (P7)
	Hospitalization facilitation	Hospitalization facilitated information gathering	“We understand sometimes it might be hard with the hospital since a lot of things are happening all at once. They’ve made it as easy as possible … [The hospitalization] was an education.” (P11)
Barriers to information gathering post-hospitalization	Barriers to outpatient cancer care	Delay in appointment with outpatient oncologist	“I’m sure they have an algorithm that decides what they do. For some people, it goes on. But in our case, seven weeks was interminable.” (C2)
		Medical bureaucracy impedes seeing outpatient oncologist	“When are we going to see the [outpatient oncologist]? Instead of just like, you’re in the hospital so he doesn’t come. And no, you haven’t done this yet? They haven’t done that yet. It’s the process. I have to say, the process is very bureaucratic. And not necessarily in the patient’s best interest.” (C2)
		Difficulty obtaining adequate supportive care to make outpatient visit	“The vendor said he will deliver the tech to his office and he will bring it down to [room] 505 and I should wait there at three o’clock. I waited until 5. He never got the tank and he [case manager] refused to answer my calls because he apparently told the guy my brother’s name and didn’t tell him enough information that the guy went to the unit where my brother had already been discharged three days ago. So, the guy went home. I was furious. We have no oxygen. There’s no way my brother can make the trip [to his outpatient visit] without oxygen.” (C2)
	Information content barrier	Lack of information integration and synthesis at discharge	“We got all of this information and these tests but nothing really came together…So, there really wasn’t a pathway to treatment. It was so scattered.” (C2) “We had kind of a goal of doing it the way we’re going to do it [in the hospital], but it was unclear if we would be able to do it that way.” (P7)
	Access to oncology care	Uncertainty in continuity of care	"She had found the health insurance confusing. Because UCSF is actually not in her network. She just happened to be admitted to the emergency room, which is how she ended up there. And so, after getting discharged, the insurance was the confusing part to try to see how we could continue care at UCSF.” (C3)
Facilitators to information gathering post-hospitalization	Access to oncology care	Plan on meeting with outpatient oncologist	“I plan on getting more information about the treatment from the people that performed my surgery. The people who give the chemo, they have the roadmap. (P9) “Yeah, when we’re actually sitting down with the oncologists and he tells us what his idea and what his plans are, then we’re going to be able to ask him more specific questions.” (C2)
Decision-making facilitators	Hospitalization facilitated decision-making	Hospitalization reduced decisional conflict	“[The hospitalization] has taken some of the pressure and anxiety away. [I went] from not knowing to having some idea of where I am, where my body is, and what the possibilities and my future existence are.” (P1)
	Home and rehabilitation better facilitate decision-making compared to being hospitalized	Being relaxed at home helps with decision-making	“I’m kind of in a much more relaxed mode being at home. I’m resting better… just to go to bed at night and can rest comfortably without any interruptions.” (P11)
		Being at home or undergoing rehabilitation helps prepare to make decisions	“[After being at home from the hospital], I’m in a better position [to make the anticipated decision].” (P2) “[In rehabilitation] I’m eating all the right foods and preparing for what I am about to go through.” (P9)

#### Anticipated cancer treatment decisions

Patients and caregivers reported that the most important anticipated cancer decision involved treatment: whether to receive treatment, treatment type, and how aggressively to treat cancer. Patients described experiencing decisional conflict due to insufficient information about the cancer, pending test results from hospitalization, and not understanding potential treatment effects.

[Anticipated decisions about the cancer] are what I gotta figure out. How can I know the future unless I know what the potential is?…I gotta know what it is before I can even think about it. (P2)

#### Variable control preferences in decision-making

Decision-making control preferences varied from self-, caregiver-, or provider-directed decisions. Most patients desired full autonomy, while some deferred decisions to their future oncologist. Two patients relied on family to make decisions due to impaired decision-making capacity from illness severity, including one patient recovering from emergent diverting colostomy surgery.

[Caregiver’s brother] and I have been making the decisions for her since she has been too sick to be able to. (C1)

#### High cancer-related information needs and uncertainty

After hospital discharge, patients and caregivers reported known and unknown information needs. Some expressed uncertainty and anxiety about their cancer care both during and after the hospitalization. One caregiver described their worries from not knowing the cancer type or treatment options during a prolonged hospitalization.

We stayed in the hospital for four or five days and nothing happened. Nobody told us what it was and nobody told us we were going to start treatment. We’re just sitting there worrying we’re going to die because we’re not going to get treatment. (C2)

Participants desired to learn about the nature of the cancer; treatment details about options, benefits, risks, and urgency; and symptom management. Prognostic information was also highly desired, such as anticipated disease course and curability. Sometimes, patients and caregivers struggled to understand why test results were delayed. One caregiver stated it would have been helpful to be informed of the reasons for the delay of biopsy results. Lastly, patients described a lack of understanding of the next steps in cancer care after discharge.

#### Barriers to information gathering during hospitalization

High information quantity and complexity, along with a lack of information due to pending or delayed test results, hindered patients’ ability to understand their cancer. The flow of information also posed a barrier. For instance, one patient who was transferred from another hospital was confused after receiving contradictory diagnoses from different hospitals about whether their cancer was small-cell or non-small-cell lung cancer. Patients also reported difficulty feeling informed due to the dynamic, rapidly collected nature of the information being gathered, synthesized, and presented to them in the hospital.

Everybody was still learning. … And they say we’ll just keep passing the information as it comes to us. … And each day was a little bit different. (P4)

There were also multiple inpatient providers delivering information, which made it difficult for patients to track and reconcile information from multiple sources.

Caregiver: When you had lots of different doctors visiting, did you know who all of them were and what they were asking you? Patient: Sort of. Some I might know; some I don't know. (C3 & P3)

Additionally, emotional distress from the cancer diagnosis and physical distress from cancer-related symptoms made it difficult for patients to absorb information in the hospital.

[In the hospital], I was just in a lot of pain and under a lot of medication and had difficulty learning some new skills as to how to navigate this new body. (P5)

#### Facilitators to information gathering during hospitalization

A number of factors helped patients gather cancer-related information in the hospital, including rapid communication of test results and availability of hospital providers to share information. Caregivers helped explain information, especially for patients whose illness compromised information processing.

Without our sister being able to explain all of the tests, I think it would have been a lot more difficult...Our sister is a doctor and is able to read her chart. She can relay that information, and that helped a lot. (C3)

The hospitalization itself, which expedited cancer diagnostic work-up, was also viewed as educational.

We understand sometimes it might be hard with the hospital since a lot of things are happening all at once. They’ve made it as easy as possible … [The hospitalization] was an education. (P11)

#### Barriers to information gathering post-hospitalization

Patients continued to face challenges in gathering and processing information after discharge but before seeing an outpatient oncologist. Some had difficulty integrating the tremendous amount of scattered data obtained during the hospitalization. Moreover, patients and caregivers faced delays in scheduling an appointment quickly with an outpatient oncologist, sometimes due to insurance coverage problems.

After getting discharged, the insurance was the confusing part to try to see how we could continue care at UCSF. (C3)

#### Facilitators to information gathering post-hospitalization

The primary means by which patients sought to obtain more information about cancer treatment after being discharged was by quickly meeting with an outpatient oncologist. Patients also sought information from other sources, such as the Internet, friends, and other physicians.

#### Decision-making facilitators

Multiple factors helped patients prepare to make cancer treatment decisions beyond the educational benefits of obtaining information. One patient said the hospitalization gave him a better sense of the cancer and future directions, which reduced his distress.

[The hospitalization] has taken some of the pressure and anxiety away. [I went] from not knowing to having some idea of where I am, where my body is, and what the possibilities and my future existence are. (P1)

For other patients, returning home to a more comfortable environment after being in the hospital helped them prepare for their anticipated cancer decisions.

### Oncologists: quantitative results

**Fig. 1:**
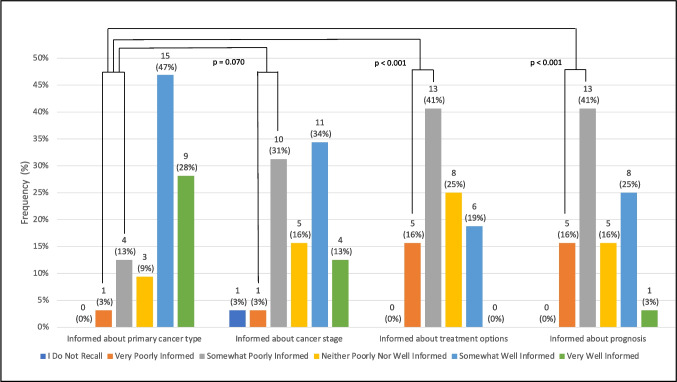
Oncologists’ perspectives on cancer-related knowledge of their most recent patient with advanced cancer newly diagnosed in the hospital. This bar graph illustrates how well-informed oncologists viewed their most recent patient with advanced cancer newly diagnosed in the hospital. From left to right, the domains of cancer information are cancer type, cancer stage, treatment options, and prognosis. On pairwise comparison to cancer type, more oncologists reported that patients were very or somewhat poorly informed about treatment options and prognosis compared to cancer type, but not about cancer stage versus cancer type

Of 46 oncologists contacted, 32 (70%) with a median 6 years of clinical experience (range 1–40 years) completed the survey. When asked about their last patient with a newly diagnosed advanced cancer in the hospital seen in their clinic, 18 (56%) oncologists reported that patients were somewhat or very poorly informed about treatment options, greater than the 5 (16%) for cancer type (*p* < 0.001; Fig. 1). The proportion for cancer prognosis (*n* = 18, 56%) was also greater than cancer type (*p* < 0.001). There was no difference in the proportion of oncologists who reported patients were somewhat or very poorly informed about cancer type versus cancer stage (*n* = 11, 34%; *p* = 0.07).

### Oncologists: qualitative results

Open-ended survey responses, with oncologist quotations indicated below by “O,” revealed themes describing cancer-related information and setting expectations regarding patients’ cancer and care plan.

#### Variable expectations about patient knowledge after hospitalization

Prior to the initial oncology appointment, oncologists reported that patients should know their cancer type, stage, and treatment (general categories, types of systemic therapy, toxicities, and curative versus palliative intent). Some reported that prognosis, such as likelihood of cure and longevity, should be disclosed early to facilitate information consolidation and outpatient consultation. Techniques to disclose prognostic information were described, such as best case-worst case framing and giving “broad strokes” ranges of survival.

It is super helpful for patients to know high level “maybe curable” or “probably not curable” so that they are emotionally prepared to listen and ask the right questions. If they hear this for the first time at clinic visit, there is often a “deer in the headlights” effect that interferes with productive consultation. (O5)

Other oncologists did not expect patients to know much about the cancer.

I’m okay with my patients having a very blank slate that I can fill in. (O6).

One oncologist believed treatment or prognostic information in the hospital is not helpful because it is frequently incorrect.

I don’t think treatment or prognosis information is helpful as the information they receive in the hospital is frequently inaccurate for their specific cancer subtype. (O3)

#### Facilitators to patient decision-making

Many oncologists wrote that inpatient oncology education is crucial in promoting SDM in the clinic, as repetition helps patients absorb and consolidate important information.

Patients often need to hear this information multiple times—it is so overwhelming at first, particularly if they are hearing it while unwell in the hospital. If they can come away knowing their diagnosis, that it is metastatic and what this means, and that there are treatment options, I think this is helpful. (O11)

Oncologists also recommended that inpatient providers highlight key cancer information in patients’ discharge instructions, such as cancer type, stage, and prognosis, provide reputable sources of online information (and what is unreliable), explain the roles of academic and community cancer centers regarding clinical trials and treatment location; describe the different roles of the multidisciplinary cancer care team, advise on the nature of the initial outpatient oncology visit, and suggest they bring in a trusted family member for support to oncology appointments.

Would be ideal to have [cancer type, stage, and rough prognosis] written down for them in discharge paperwork because patients often forget when they see doctors early AM/late PM/without family around/right after procedural sedation/etc. (O5)

To help outpatient oncologists communicate with these patients, they recommended inpatient providers document what information was or was not discussed with patients in the hospital, or give a handoff that includes pertinent inpatient data, such as inpatient care received, what information was given to the patient, and pending test results. Lastly, systemic changes to facilitate timely establishment of outpatient care were mentioned.

#### Patient uncertainty before outpatient oncologist appointment

Oncologists also acknowledged the significant uncertainty patients face while waiting for their outpatient oncology appointment following hospital discharge.

## Discussion and conclusion

### Discussion

In this mixed-methods study of patients, caregivers, and oncologists, we found that patients with advanced cancer newly diagnosed in the hospital have numerous information and decisional needs, experience decisional conflict, and face barriers to fulfilling these needs, many of which are unique to or exacerbated by hospitalization.

The interviews and surveys revealed insights on these patients’ information and decisional needs prior to meeting their outpatient oncologist. We found that typically, patients had an idea of their cancer type and stage, but most were unclear about treatment and prognosis, which is supported by our oncologist survey results and findings reported in the outpatient setting [14]. This cascade of knowledge follows the order of operations of first diagnosing and staging the cancer and then determining treatment and prognosis. Patients’ knowledge gaps about treatment and prognosis manifested as decisional conflict and uncertainty, which oncologists empathized as challenging for patients to experience as they await meeting their future oncologist.

Known barriers to information gathering and processing that were exacerbated by hospitalization contributed to these knowledge gaps. Barriers included emotional and physical distress from patients’ illness and low recall of complex, voluminous information, consistent with findings in studies in similar contexts [15–19]. Notably, patients experienced uncertainty due to inconsistencies in diagnostic test results as well as suboptimal provider-patient communication due to both a lack of information as well as the relaying of complex information about their cancer, which has not been previously reported in this context to our knowledge. The way such data were presented in the hospital was also uniquely challenging, such as difficulty reconciling information from numerous and constantly changing providers and lack of integration of inpatient test results following discharge. Lastly, all stakeholders recognized that establishing timely outpatient care was important in promoting SDM surrounding cancer treatment.

Despite the challenges of hospitalization, all participants described that the hospitalization also presented an opportunity to address patients’ information and decisional needs and aforementioned barriers. Beyond the benefits of rapid data gathering and reporting, hospitalization also alleviated decisional uncertainty since information can be relayed quickly and questions answered immediately by inpatient providers. This adds to previous studies that found that the ability to exchange desired information facilitates SDM in hospitalized patients [18, 20–22]. Oncologists also described how hospitalization can promote information retention and consolidation since inpatient providers can start a chain of information repetition, preparing patients to participate in SDM [23, 24].

Potential remedies to overcome the aforementioned barriers were identified. Consistent with what is known for patients with advanced cancer in the outpatient setting [8], actively involving caregivers was helpful in not only obtaining and consolidating information but also surrogating decision-making if needed. Oncologists recommended handoffs and tailored, standardized information in discharge instructions, which could address difficulties regarding pending test follow-up and a lack of information integration at discharge. This complements and adds to a recent qualitative oncology inpatient SDM study which proposed that the electronic health record could be leveraged to better promote SDM [18]. Lastly, education on reputable online information and orientation to cancer care delivery were recommended by both patients and oncologists to facilitate effective SDM.

There was a general concordance between patients and oncologists on the type of information patients ideally should know following discharge. However, there was concern for inaccuracy in treatment and prognostic information patients received in the hospital. This concern may have arisen from difficulty in providing accurate treatment and prognostic information with incomplete information and that patients’ performance status while hospitalized may not reflect their true baseline [25]. Also, inpatient providers are usually general oncologists who may have insufficient knowledge to educate patients on rare malignancies. For most patients, we suggest that inpatient providers communicate treatment and prognostic information in broad strokes based on available information, with contingencies based on pending information. This is because repetition of serious news may help information retention and increase emotional preparedness during treatment SDM. Furthermore, earlier prognosis disclosure and awareness of incurability of their cancer are associated with more accurate prognosis awareness and patient-directed decisions like hospice enrollment, respectively [23, 24]. Attention should be paid to prognostic subgroups that may vary based on pending tests, such as molecular testing, and it should be conveyed that this prognostic uncertainty will be clarified with their outpatient oncologist.

The single-center design, few caregivers, and exclusion of non-English-speaking patients limit the generalizability of our findings. Our study also did not explore patient preferences, which are important in SDM. Nonetheless, the mixed-methods design across three key decision-making roles and high patient and oncologist response rates strengthen our findings.

### Conclusion

Patients diagnosed with a new advanced cancer in the hospital have numerous SDM needs, mainly surrounding treatment and prognostic information, and face decisional conflict surrounding anticipated treatment decisions. Barriers to SDM can be exacerbated by hospitalization, since acute emotional and physical distress limit cancer information absorption, and information is often high in volume and complexity and delivered by multiple, constantly changing providers. Despite these barriers, patients may become better prepared for SDM regarding their cancer because they have a concentrated time for information-sharing with providers and to quickly learn about their cancer, alleviating uncertainty and anxiety. Further investigation is needed to test potential interventions, such as those identified by our stakeholders, to address barriers and augment facilitators. Doing so may enhance SDM and promote high-quality cancer care for this vulnerable population.

### Supplementary Information

Below is the link to the electronic supplementary material.

Supplementary file1 (DOCX 52.3 KB)

## Data Availability

The data that support the findings of this study are available upon request from the corresponding author.

## References

[1] Elwyn G, Frosch D, Thomson R, Joseph-Williams N, Lloyd A, Kinnersley P, Cording E, Tomson D, Dodd C, Rollnick S, Edwards A, Barry M (2012). Shared decision making: a model for clinical practice. J Gen Intern Med.

[2] Ganz PA (2014). Institute of Medicine report on delivery of high-quality cancer care. J Oncol Pract.

[3] Hower EG (2020). Beyond shared decision making. J Clin Ethics.

[4] Lewandowska A, Rudzki G, Lewandowski T, Rudzki S (2020) The problems and needs of patients diagnosed with cancer and their caregivers. Int J Environ Res Public Health 18. 10.3390/ijerph18010087 10.3390/ijerph18010087 PMC7795845 33374440

[5] Mazzocco K, Masiero M, Carriero MC, Pravettoni G (2019). The role of emotions in cancer patients’ decision-making. Ecancermedicalscience.

[6] Wang T, Molassiotis A, Chung BPM, Tan J-Y (2018). Unmet care needs of advanced cancer patients and their informal caregivers: a systematic review. BMC Palliat Care.

[7] Delamare Fauvel A, Bischof JJ, Reinbolt RE, Weihing VK, Boyer EW, Caterino JM, Wang HE (2023) Diagnosis of cancer in the Emergency Department: a scoping review. Cancer Med. 10.1002/cam4.5600 10.1002/cam4.5600 PMC10134283 36622062

[8] Dionne-Odom JN, Ejem D, Wells R, Barnato AE, Taylor RA, Rocque GB, Turkman YE, Kenny M, Ivankova NV, Bakitas MA, Martin MY (2019). How family caregivers of persons with advanced cancer assist with upstream healthcare decision-making: a qualitative study. PLoS ONE.

[9] van Vliet LM, Meijers MC, van Dulmen S, van der Wall E, Plum N, Stouthard J, Francke AL (2021). Addressing challenges in information-provision: a qualitative study among oncologists and women with advanced breast cancer. BMC Palliat Care.

[10] Tong A, Sainsbury P, Craig J (2007). Consolidated criteria for reporting qualitative research (COREQ): a 32-item checklist for interviews and focus groups. Int J Qual Health Care.

[11] Jacobsen MJ, O’Connor AM, Stacey D (2013) Decisional needs assessment in populations a workbook for assessing patients’ and practitioners’ decision making needs, Ottowa

[12] Francis JJ, Johnston M, Robertson C, Glidewell L, Entwistle V, Eccles MP, Grimshaw JM (2010). What is an adequate sample size? Operationalising data saturation for theory-based interview studies. Psychol Health.

[13] Fetters MD, Curry LA, Creswell JW (2013). Achieving integration in mixed methods designs—principles and practices. Health Serv Res.

[14] Rutten LJF, Arora NK, Bakos AD, Aziz N, Rowland J (2005). Information needs and sources of information among cancer patients: a systematic review of research (1980–2003). Patient Educ Couns.

[15] Gabrijel S, Grize L, Helfenstein E, Brutsche M, Grossman P, Tamm M, Kiss A (2008). Receiving the diagnosis of lung cancer: patient recall of information and satisfaction with physician communication. J Clin Oncol.

[16] Kessels RPC (2003). Patients’ memory for medical information. J R Soc Med.

[17] Laws MB, Lee Y, Taubin T, Rogers WH, Wilson IB (2018). Factors associated with patient recall of key information in ambulatory specialty care visits: results of an innovative methodology. PLoS One.

[18] Steenbergen M, de Vries J, Arts R, Beerepoot LV, Traa MJ (2022). Barriers and facilitators for shared decision-making in oncology inpatient practice: an explorative study of the healthcare providers’ perspective. Support Care Cancer.

[19] Ekdahl AW, Andersson L, Wiréhn A-B, Friedrichsen M (2011). Are elderly people with co-morbidities involved adequately in medical decision making when hospitalised? A cross-sectional survey. BMC Geriatr.

[20] Mohan D, Alexander SC, Garrigues SK, Arnold RM, Barnato AE (2010). Communication practices in physician decision-making for an unstable critically ill patient with end-stage cancer. J Palliat Med.

[21] Gualano MR, Bert F, Passi S, Stillo M, Brescia V, Scaioli G, Thomas R, Voglino G, Minniti D, Boraso F, Siliquini R (2019). Could shared decision making affect staying in hospital? A cross-sectional pilot study. BMC Health Serv Res.

[22] Decker C, Garavalia L, Chen C, Buchanan DM, Nugent K, Shipman A, Spertus JA (2007). Acute myocardial infarction patients’ information needs over the course of treatment and recovery. J Cardiovasc Nurs.

[23] Liu P-H, Landrum MB, Weeks JC, Huskamp HA, Kahn KL, He Y, Mack JW, Keating NL (2014). Physicians’ propensity to discuss prognosis is associated with patients’ awareness of prognosis for metastatic cancers. J Palliat Med.

[24] Mack JW, Walling A, Dy S, Antonio ALM, Adams J, Keating NL, Tisnado D (2015). Patient beliefs that chemotherapy may be curative and care received at the end of life among patients with metastatic lung and colorectal cancer. Cancer.

[25] Hochman MJ, Wolf S, Zafar SY, Portman D, Bull J, Kamal AH (2016). Comparing unmet needs to optimize quality: characterizing inpatient and outpatient palliative care populations. J Pain Symptom Manage.

