# Supplementary material for: Shared decision-making needs, barriers, and facilitators of patients with newly diagnosed advanced cancer in the hospital: a multi-level, mixed-methods study
Source: Support Care Cancer. 2024 Apr 29;32(5):315. doi: 10.1007/s00520-024-08515-1 (PMC11058864; doi:10.1007/s00520-024-08515-1)
Supplement: Supplementary file 1 — Supplementary file1 (DOCX 52.3 KB) [file 520_2024_8515_MOESM1_ESM.docx]

Shared decision-making needs, barriers, and facilitators of patients with newly diagnosed advanced cancer in the hospital: a multi-level, mixed-methods study

Journal: Supportive Care in Cancer

Kevin R. Reyes^1^, BS; Paul Wong^1^, BS; Mary Petrofsky^2^, MS, RN, ACNP-BC, AOCNP; Annie Dai^2^, BA; Alyson Pelayo^2^, MSN, RN, FNP-C; Sam Brondfield^1,2^, MD, MAEd; Daniel H. Kwon^1,2^, MD

1. Department of Medicine, University of California, San Francisco (UCSF), San Francisco, CA, USA.

2. UCSF Helen Diller Family Comprehensive Center, University of California, San Francisco (UCSF), San Francisco, CA, USA.

Corresponding author email: [daniel.kwon@ucsf.edu](mailto:daniel.kwon@ucsf.edu)

**Supplementary Table 1: Consolidated criteria for reporting qualitative studies (COREQ) 32-item checklist**

| **No. Item** | **Guide questions** | **Description** |
| --- | --- | --- |
| **Domain 1: Research team and reﬂexivity** | | |
| Personal Characteristics | | |
| 1. Interviewer/facilitator | Which author/s conducted the interview or focus group? | Kevin R. Reyes  Paul Wong |
| 2. Credentials | What were the researcher’s credentials? E.g. PhD, MD | Kevin R. Reyes: BS  Paul Wong: BS  Mary Petrofsky: MS, RN, ACNP-BC, AOCNP  Annie Dai: BA  Alyson Pelayo: MSN, RN, FNP-C  Sam Brondfield: MD, MAEd  Daniel H. Kwon: MD |
| 3. Occupation | What was their occupation at the time of the study? | Kevin R. Reyes: Medical student  Paul Wong: Medical student  Mary Petrofsky: Nurse Practitioner  Annie Dai: Inpatient practice coordinator  Alyson Pelayo: Nurse Practitioner  Sam Brondfield: Hospital-based oncologist  Daniel H. Kwon: Hospital-based oncologist |
| 4. Gender | Was the researcher male or female? | Kevin R. Reyes: Male  Paul Wong: Male  Mary Petrofsky: Female  Annie Dai: Female  Alyson Pelayo: Female  Sam Brondfield: Male  Daniel H. Kwon: Male |
| 5. Experience and training | What experience or training did the researcher have? | Kevin R. Reyes: Academic researcher, medical student, completed interview training with Daniel H. Kwon.  Paul Wong: Academic researcher, medical student, completed interview training with Daniel H. Kwon.  Mary Petrofsky: None  Annie Dai: None  Alyson Pelayo: None  Sam Brondfield: Academic researcher with a focus on qualitative research in medical education. MAEd included qualitative research methods.  Daniel H. Kwon: Academic researcher with a focus on mixed-methods research in shared-decision making for patients with cancer. Completed formal course work in qualitative research methods. |
| Relationship with participants | | |
| 6. Relationship established | Was a relationship established prior to study commencement? | Yes. Participants were under the care of at least one member of the inpatient oncology team (M.P., A.D., A.P., S.B., and/or D.H.K.). |
| 7. Participant knowledge of the interviewer | What did the participants know about the researcher? e.g. personal goals, reasons for doing the research | Participants knew that the goal of the researcher was to improve the decision-making of patients with cancer when they meet their future outpatient oncologist. |
| 8. Interviewer characteristics | What characteristics were reported about the interviewer/facilitator? e.g. Bias, assumptions, reasons and interests in the research topic | The research team works predominantly in the inpatient setting (aside from D.H.K. who also has an outpatient oncology practice), which may have created biases and assumptions of participants. |
| **Domain 2: study design** | | |
| Theoretical framework | | |
| 9. Methodological orientation and Theory | What methodological orientation was stated to underpin the study? e.g. grounded theory, discourse analysis, ethnography, phenomenology, content analysis | Ottawa Decision Support Framework: Conceptualizes the support needed by patients, families, and providers for difficult decisions |
| Participant selection | | |
| 10. Sampling | How were participants selected? e.g. purposive, convenience, consecutive, snowball | Patients were consecutively sampled among inpatients for whom medical oncology consultation was requested. |
| 11. Method of approach | How were participants approached? e.g. face-to-face, telephone, mail, email | Face-to-face |
| 12. Sample size | How many participants were in the study? | Twelve patients, 4 caregivers, and 32 oncologists. |
| 13. Non-participation | How many people refused to participate or dropped out? Reasons? | Six patients did not participate, of whom 3 (50%) were too distressed to participate, 2 (33%) passed away before the interview could be conducted, and 1 (17%) declined to provide a reason. |
| Setting | | |
| 14. Setting of data collection | Where was the data collected? e.g. home, clinic, workplace | Survey data were collected in the patient’s hospital room or online (at the patient’s residence).  Interview data were collected by phone, with all parties at their own residence. |
| 15. Presence of non-participants | Was anyone else present besides the participants and researchers? | No |
| 16. Description of sample | What are the important characteristics of the sample? e.g. demographic data, date | See Table 1 |
| Data collection | | |
| 17. Interview guide | Were questions, prompts, guides provided by the authors? Was it pilot tested? | See Supplementary figure 2. This guide was pilot tested and revised for patient comprehension. |
| 18. Repeat interviews | Were repeat interviews carried out? If yes, how many? | No |
| 19. Audio/visual recording | Did the research use audio or visual recording to collect the data? | Interview data were collected using audio recording. |
| 20. Field notes | Were ﬁeld notes made during and/or after the interview or focus group? | No |
| 21. Duration | What was the duration of the interviews or focus group? | Thirty minutes on average. |
| 22. Data saturation | Was data saturation discussed? | Following initial analysis set at a sample size of 8, the pre-specified stopping criteria for saturation was defined as no new themes in two consecutive interviews |
| 23. Transcripts returned | Were transcripts returned to participants for comment and/or correction? | No |
| **Domain 3: analysis and ﬁndings** | | |
| Data analysis | | |
| 24. Number of data coders | How many data coders coded the data? | Two (K.R.R., P.W.) |
| 25. Description of the coding tree | Did authors provide a description of the coding tree? | Codes were categorized into subthemes and themes by K.R.R. and D.H.K (8 themes, 25 subthemes). |
| 26. Derivation of themes | Were themes identiﬁed in advance or derived from the data? | Themes were identified in advance using the interview guide, and emergent themes were added or modified during the coding process. |
| 27. Software | What software, if applicable, was used to manage the data? | Microsoft Word and ATLAS.ti. |
| 28. Participant checking | Did participants provide feedback on the ﬁndings? | No |
| Reporting | | |
| 29. Quotations presented | Were participant quotations presented to illustrate the themes/ﬁndings? Was each quotation identiﬁed? e.g. participant number | Yes. Participants were identified by category (patient, caregiver, oncologist) and assigned number. |
| 30. Data and ﬁndings consistent | Was there consistency between the data presented and the ﬁndings? | Yes |
| 31. Clarity of major themes | Were major themes clearly presented in the ﬁndings? | Yes. Eight major themes are reported in this publication. |
| 32. Clarity of minor themes | Is there a description of diverse cases or discussion of minor themes? | Yes. Divergent cases were reported, e.g., one oncologist did not feel it was helpful for patients to receive treatment or prognostic information in the hospital. |

**Supplementary Figure 1: Patient Survey**

Our goal is to understand the decision-making needs of patients who are newly diagnosed with cancer in the hospital. A better understanding of these needs will help us develop tools that help other patients make well-informed decisions about their cancer. In this survey and a future interview, we want to learn about your personal perspectives on the information and support you need.

Please answer the following questions to the best of your ability.

Basic knowledge of the cancer

Do you know the type of cancer you have?

Type means the part of the body where the cancer started

**▢** Yes

**▢** No

Do you know the stage of the cancer you have?

Stage means how far the cancer has spread

**▢**Yes

**▢**No

Decisional conflict scale

Considering treatment options for the cancer, please answer how much you agree or disagree right now with the following statements:

|  | Strongly Agree | Agree | Neither agree nor disagree | Disagree | Strongly disagree |
| --- | --- | --- | --- | --- | --- |
| I know which options are available to me | **▢** | **▢** | **▢** | **▢** | **▢** |
| I know the benefits of each option | **▢** | **▢** | **▢** | **▢** | **▢** |
| I know the risks and side effects of each option | **▢** | **▢** | **▢** | **▢** | **▢** |
| I am clear about which benefits matter most to me | **▢** | **▢** | **▢** | **▢** | **▢** |
| I am clear about which risks and side effects matter most to me | **▢** | **▢** | **▢** | **▢** | **▢** |
| I am clear about which is more important to me (the benefits or the risks and side effects) | **▢** | **▢** | **▢** | **▢** | **▢** |
| I have enough support from others to make a choice | **▢** | **▢** | **▢** | **▢** | **▢** |
| I am choosing without pressure from others | **▢** | **▢** | **▢** | **▢** | **▢** |
| I have enough advice to make a choice | **▢** | **▢** | **▢** | **▢** | **▢** |
| I am clear about the best choice for me | **▢** | **▢** | **▢** | **▢** | **▢** |
| I feel sure about what to choose | **▢** | **▢** | **▢** | **▢** | **▢** |
| This decision is easy for me to make | **▢** | **▢** | **▢** | **▢** | **▢** |

Demographics

All the following demographic questions are optional.

What is your age?

On your original birth certificate, were you listed as male or female?

**▢**Male

**▢**Female

What is your current gender identity?

**▢**Male

**▢**Female

**▢**Transgender

**▢**Gender non-conforming

**▢**Other – Specify

Which of the following best describes your current occupational status?

Mark all that apply.

**▢**Employed

**▢**Unemployed for 1 year or more

**▢**Unemployed for less than 1 year

**▢**Homemaker

**▢**Student

**▢**Retired

**▢**Disabled

**▢**Other-Specify

What is your marital status?

Mark only one.

**▢**Married

**▢**Living as married or living with a romantic partner

**▢**Divorced

**▢**Widowed

**▢**Separated

**▢**Single, never been married

What is the highest grade or level of schooling you completed?

Mark only one.

**▢**Less than 8 years

**▢**8 through 11 years 12 years or completed high school

**▢**Post high school training other than college (vocational or technical)

**▢**Some college

**▢**College graduate

**▢**Postgraduate

Are you of Hispanic, Latino/a, or Spanish origin?

**▢**Yes

**▢**No

What is your race? One or more categories may be selected. Mark all that apply

**▢**White

**▢**Black or African American

**▢**American Indian or Alaska Native

**▢**Asian Indian

**▢**Chinese

**▢**Filipino

**▢**Japanese

**▢**Korean

**▢**Vietnamese

**▢**Other Asian

**▢**Native Hawaiian

**▢**Guamanian or Chamorro

**▢**Samoan

**▢**Other Pacific Islander

**▢**Other – specify

Including yourself, how many people live in your household?

Thinking about members of your family living in this household, what is your combined annual income, meaning the total pre-tax income from all sources earned in the past year?

**▢**$0 to $9,999

**▢**$10,000 to $14,999

**▢**$15,000 to $19,999

**▢**$20,000 to $34,999

**▢**$35,000 to $49,999

**▢**$50,000 to $74,999

**▢**$75,000 to $99,999

**▢**$100,000 to $199,999

**▢**$200,000 or more

**Supplementary Figure 2: Patient Caregiver Interview Guide**

The topic of today’s interview is helping patients with newly diagnosed cancer in the hospital make future decisions about the cancer. Informed decision-making is a process in which patients are given information about their cancer and are able to understand and process the information. The goal is to ensure that patients make informed decisions about the cancer when they meet their future oncologist, or cancer doctor. We are working with the doctors and nurses here to evaluate how well patients are informed and what we can do to better support them. The information that you share in this interview will help us. Please remember there are no right or wrong answers. We want to learn about your personal experience.

1. What have you been told about the (suspected) cancer?
2. What decisions do you anticipate making about the (suspected) cancer in the near future? I am most interested in hearing about medical decisions and not administrative decisions, like scheduling appointments.

[Summarize and restate decisions prior to asking question]

1. Which of these decisions is the most important to you?

[If doesn’t have one or is confused, prompt the participant:

Here are some decisions that other people in a similar situation have found important:

Should I undergo treatment for the cancer or not?

What type of treatment should be started?

Should I pursue more procedures?

Let’s talk about the difficulty with making (participant’s most important decision).

1. Does this decision seem difficult or complicated to you?

- If so, what things make this decision difficult or complicated?
- Has anything else made the decision difficult or complicated?
- How has being hospitalized made the decision difficult or complicated, if at all?

1. Are you lacking information about the (suspected) cancer?

- What information are you lacking?
- Which information is the most important for you to find out?
- Why is this information the most important to you?
- Is any information that you’ve received confusing? If so, what information?
- How has being hospitalized made becoming knowledgeable about the cancer harder?

1. Thinking about (participant’s most important decision), do you know what options you have?

- [If yes]: What are all the options?
- [If no]: What has prevented you from learning about what options you have?

1. Is there anything else you wish you knew about the (suspected) cancer or its treatments?

Next, I’d like to talk about how you will go about making (participant’s most important decision).

1. How are you going to make this decision?

[If the participant is confused or doesn’t know, here are prompts]:

- Do you plan to get more information?
- What type of information?
- From where/whom?
- Will you obtain support from others?
- How will you handle and cope with any pressure associated with making this decision?

1. What do you think might get in the way of you making (participant’s most

important decision)?

- Probe and ask why/how.
- Anything else?

1. Do you think that the transition to (home/nursing facility) might make the upcoming decision-making process easier or harder?

- If yes, how so?

1. What do you think might help you overcome these barriers to making (participant’s most important decision)?

- Probe and ask why/how
- Anything else?

1. If you could have help with making (participant’s most important decision), what might that help look like?  
   [If the patient is confused or doesn’t know] Some examples of help might include counseling from a health provider, patient support groups, and online or written materials.

- Anything else?

1. This concludes the interview. Do you have any questions?

**Supplementary Figure 3: Oncologist Survey**

Our overall goal is to understand the decisional needs of patients who are newly diagnosed with advanced cancer in the inpatient setting, so we can learn how to improve their access to the information and tools needed for them to make well-informed decisions. Currently, we are studying the extent to which patients are prepared to make well-informed, preference-based decisions at their first New Patient oncology appointment. We want to learn about medical oncologists’ perspectives on these issues.

Please think back to the most recent clinic appointment you had with a patient who was just diagnosed with a metastatic cancer in the hospital. It does not matter whether this was a *de novo* metastatic presentation or a first metastatic recurrence. If you cannot recall any such patient, please imagine a composite patient based on collective prior patients.

At the start of the appointment, how informed was your patient about their...

|  | Very poorly informed | Somewhat poorly informed | Neither poorly nor well informed | Somewhat well informed | Very well informed | N/A- we did not discuss | I do not recall |
| --- | --- | --- | --- | --- | --- | --- | --- |
| Primary Cancer Type | **▢** | **▢** | **▢** | **▢** | **▢** | **▢** | **▢** |
| Cancer Stage | **▢** | **▢** | **▢** | **▢** | **▢** | **▢** | **▢** |
| Treatment options | **▢** | **▢** | **▢** | **▢** | **▢** | **▢** | **▢** |
| Prognosis | **▢** | **▢** | **▢** | **▢** | **▢** | **▢** | **▢** |

All of the following questions are optional

Now, please consider all patients you have seen in the clinic who were just diagnosed with a metastatic cancer in the hospital.

What cancer-related information do you wish these patients knew at the appointment?

What cancer-related informational resources do you wish these patients and caregivers were provided prior to their appointment?

Note: resources include educational material or others (such as a support group)

How can the inpatient solid oncology consult team better prepare these patients and providers to make well-informed, preference-based decisions when they meet with you for the first time in the clinic?
